# Evaluation of a porous hydroxyapatite alloplast in the management of grade II furcation defects in molars; A case series

**DOI:** 10.4317/jced.50726

**Published:** 2012-04-01

**Authors:** Jayan J. Mathew, M S. Arun Kumar, K M. Bhat

**Affiliations:** 1 MDS. Reader Dept of Periodontics Mar Baselios Dental College Kothamangalam Kerala; 2 MDS, MFGDP, Professor Department of Periodontics. Yenopoya Dental. College. Mangalore. India; 3 BSc, MDS. Professor, Department of Periodontics. Manipal College of Dental Sciences. Manipal, India

## Abstract

Introduction: The present case series evaluates the efficacy of a hydroxyapatite graft material in the management of grade II furcation involvement in first molars. Material and methods: Eight patients presenting with a total of 9 grade II furcation defects in relation to the facial aspect of either maxillary or mandibular first molars were treated with a porous hydroxyapatite alloplast. The clinical outcomes were measured in terms of change in probing pocket depth and clinical attachment level (vertical and horizontal) at 6 months post-operatively. Radiographs were used as supplements. Results: At 6 months, there was a mean pocket depth reduction of 3.12±1.25 mm, a mean vertical attachment gain of 2.75±1.17 mm, and a mean horizontal attachment gain of 3.25±1.28 mm. Radiographs showed bone fill at all treated sites. The outcomes were better in mandibular teeth compared to maxillary teeth. The use of hydroxyapatite graft is effective in reducing pocket depth and bringing out gain in attachment levels when used in grade II furcation defects.

** Key words:**Grade II furcation, bone grafts, hydroxyapatite.

## Introduction

Management of periodontally involved furcations of multirooted teeth has been one of the most challenging problems in periodontal therapy. The primary objective of any furcation therapy is the elimination of the pocket by resective or regenerative procedures and making the area accessible for plaque control (1). A number of studies conducted in the past two decades have shown that grade II furcation defects respond favorably to regenerative procedures in a predictable manner (2). Over the years, bone grafts have been widely used in the management of periodontal osseous defects and are still the most preferred regenerative technique (3). Hydroxyapatite is a tribasic calcium phosphate ceramic, which has been widely used as an alloplastic bone replacement material in the regeneration of periodontal defects (5). To date, very few studies have evaluated the use of hydroxyapatite grafts alone in the management of furcation involvements (5,6).The present study was undertaken to evaluate the efficacy of a hydroxyapatite graft material (Periobone G, TopNotch HealthCare Products Ltd, Aluva, India), in the management of grade II furcation involvement in first molars.

## Material and Methods

The patients for this study were selected from outpatients attending the Department of Periodontics, Manipal College of Dental Sciences, Manipal, India. The patients were selected according to the following criteria; presence of a first molar (maxillary and / or mandibular) showing a buccal grade II furcation involvement (Glickman’s classification), no proximal furcation involvement in the case of maxillary first molars, teeth free of any pulpal or periapical pathology, possibility of covering the furcation completely following flap closure, presence of no deleterious oral habits such as smoking, and presence of no contributing systemic diseases.

Eight patients fulfilling the above mentioned criteria were included in the study. There were 5 males and 3 females belonging to the age group 30 to 52 years. A total of nine grade II furcation defects (5 maxillary, 4 mandibular, 2 defects in one patient) were treated. The purpose of the study was explained to the patients and consent was obtained. After selection each patient underwent an initial preparatory phase that included oral hygiene instructions and scaling and root planing. Occlusal adjustment was performed in instances where trauma from occlusion was present.

After the completion of the initial phase, both vertical and horizontal parameters of the furcation defects were measured. The vertical parameters included probing pocket depth (PPD), and probing attachment level (PAL). The horizontal parameter measured was the horizontal attachment level (HAL). The vertical parameters were assessed using a University of Michigan “O” probe with William’s markings. Reproducible alignments of the probe were provided by custom made self cure acrylic stents grooved in an occlusoapical direction on the midbuccal aspect corresponding to the furcation. The apical border of the stent served as a fixed reference point for taking the measurements. The following measurements were recorded.

1. Reference point (RP) to gingival margin (GM)

2. Reference point (RP) to cementoenamel junction

3. (CEJ)

4. Reference point (RP) to base of the pocket (BOP)

The probing pocket depth (PPD) was calculated by noting the differences between measurements from the reference point to gingival margin to reference point to base of the pocket.

PPD = RP to BOP – RP to GM

Probing attachment level (PAL) was calculated by noting the difference between the distances reference point to base of the pocket and reference point to cementoenamel junction.

PAL = RP to BOP – RP to CEJ

The horizontal attachment levels (HAL) were assessed using a graduated color-coded Naber’s probe (PQ2N, HuFriedy Co.,) marked at 3mm increments (at 3, 6, 9, 12 mm). The buccal groove was used as a guideline for probe placement. The cementoenamel junction (CEJ) was taken as the fixed reference point for horizontal measurements.

Gingival recession (GR) was calculated by noting the difference between the distances reference point to gingi-val margin and reference point to cementoenamel junction.

GR = RP to GM – RP to CEJ

As a supplement to the clinical measurements, intraoral periapical radiographs of the defects were obtained by the long cone paralleling technique with a calibrated grid (Table  Table 1, Table 2, Table 3, Table 4).

**Table 1:**
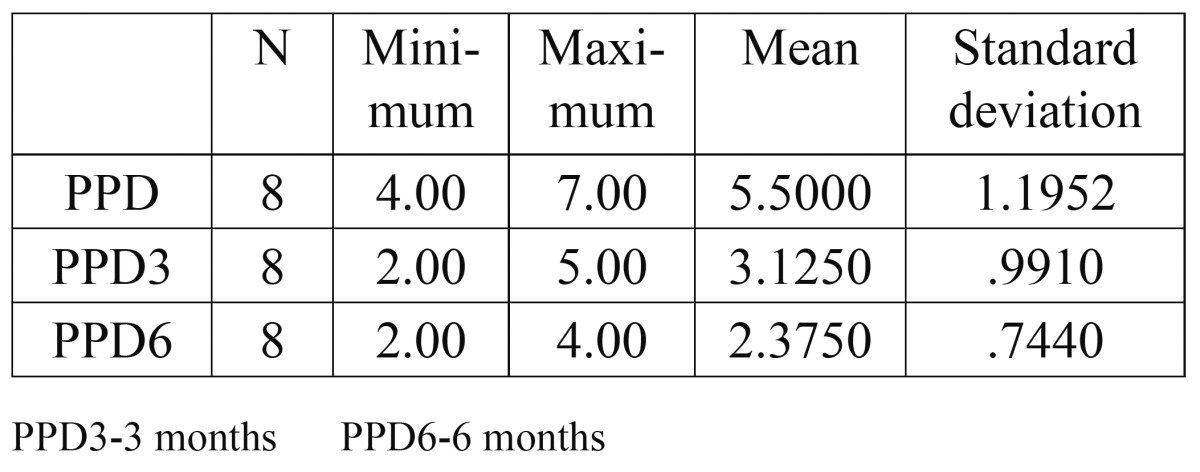
Probing Pocket Depth (PPD).

**Table 2:**
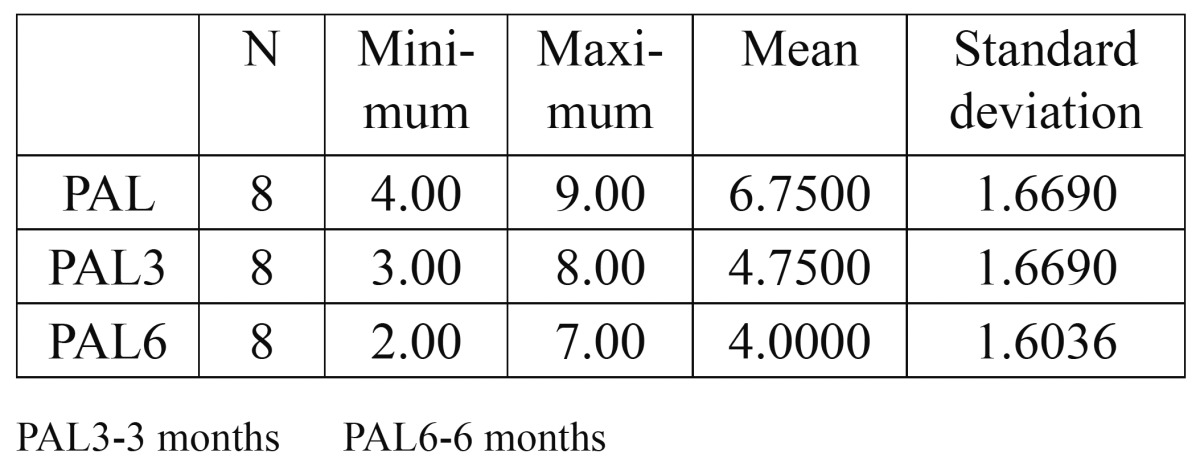
Probing Attachment Level (PAL).

**Table 3:**
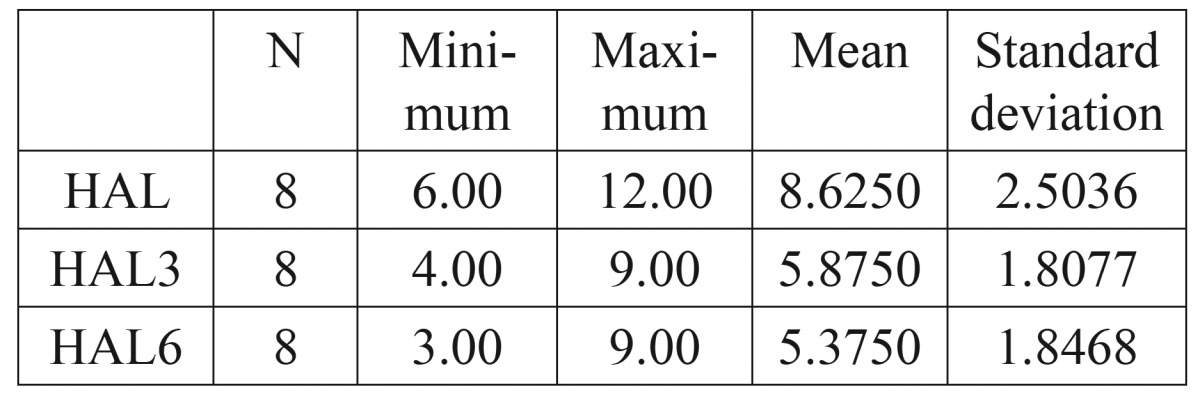
Horizontal Attachment Level (HAL).

**Table 4:**
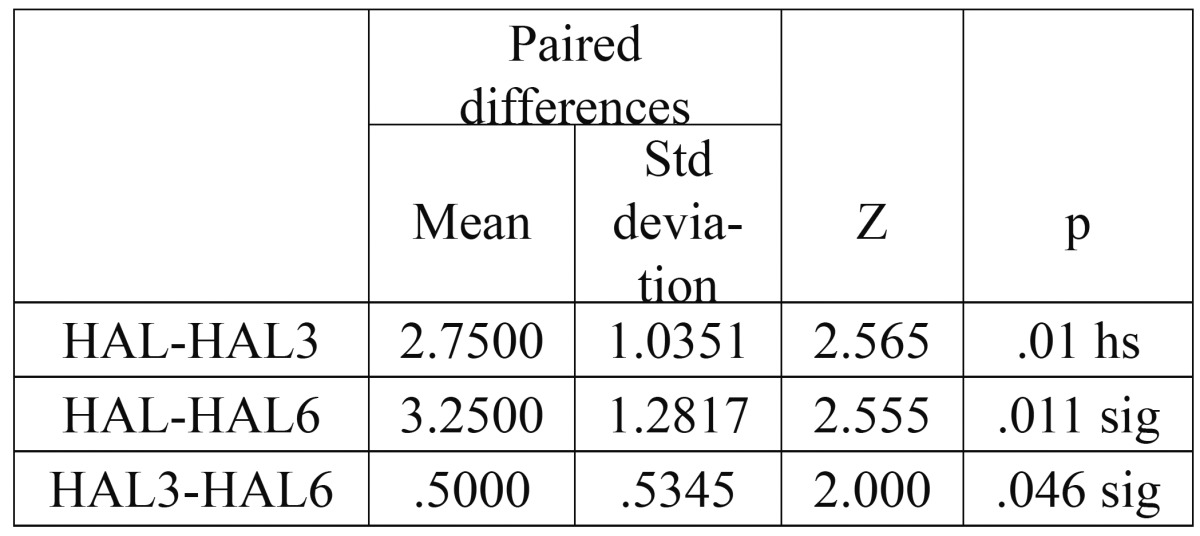
Paired samples test for Horizontal Attachment Level.

The patients were reevaluated 2-4 weeks after the initial therapy and when the patients demonstrated adequate oral hygiene maintenance and the tissues showed no clinical signs of inflammation, surgical therapy was initiated. After the area was anesthetized, a crevicular incision was given using a no.15 blade and a full thickness mucoperiosteal flap was elevated to expose the furcation defect. The furcation was thoroughly debrided using hand and ultrasonic instruments. Thorough root planing was done using curettes. The graft material was dispensed into a sterile dappen dish and mixed with sterile saline. The material was then placed into the furcation defect in increments and gently condensed using an amalgam condenser. When the defect was filled with the graft, the flap was repositioned and sutured using interrupted sutures with 30 black silk. Slight coronal repositioning of the flaps was obtained to ensure adequate coverage of the furcation, wherever required. A periodontal dressing (CoePack) was placed over the surgical site.

Post-operatively, all the patients were placed under systemic antibiotics (amoxicillin 500 mg, three times daily) for five days. Suitable anti-inflammatory analgesic agents were also prescribed. The patients were instructed to rinse twice daily with 0.2% chlorhexidine mouthwash for 2 weeks. The periodontal dressings and sutures were removed after one week. Following this, the patients were kept on maintenance care and were reviewed at 3 months and six months for evaluation.

All the preoperative measurements were repeated at 3 months and 6 months post-operatively and the changes were noted. The radiographs were also repeated at these time periods. The measurements obtained were statistically analyzed using the Wilcoxon’s signed ranks test at a significance level of 0.05.

## Results

Following surgery, all the treated sites demonstrated a normal healing pattern without any signs of infection or patient discomfort. The maintenance of oral hygiene was also satisfactory in all the patients.

**Figure 1:**
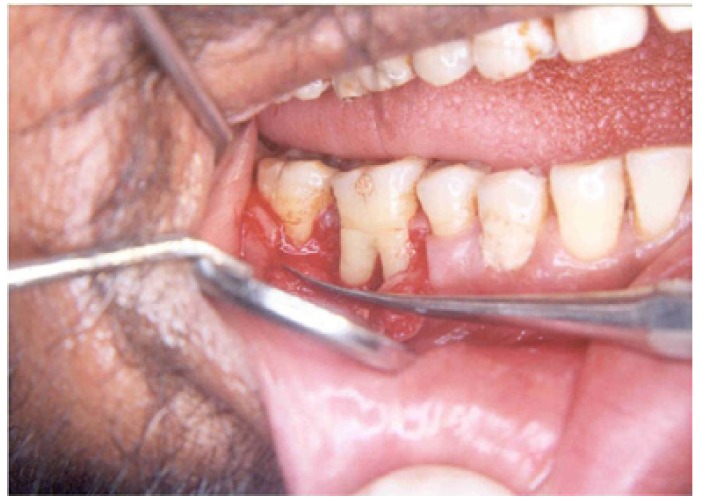
Grade II buccal furcation defect in relation to 46; after flap reflection and debridement.

**Figure 2:**
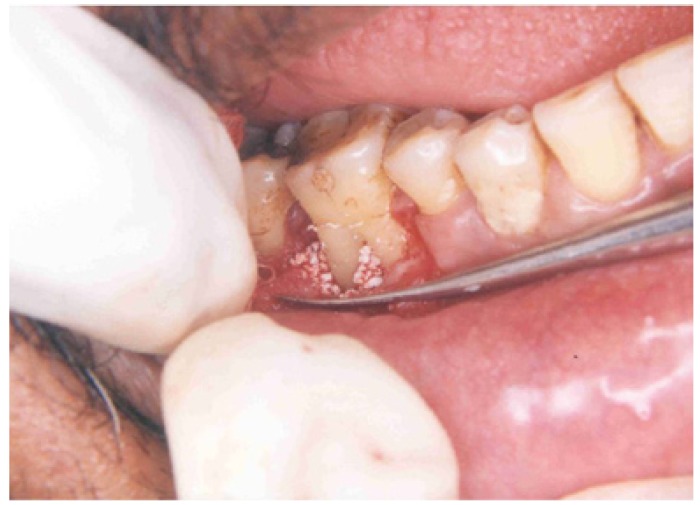
Defect filled with bone graft.

The clinical parameters, i.e., probing pocket depth, probing attachment level, horizontal attachment level, and gingival recession were assessed preoperatively and 3 months and 6 months following the surgery (Fig. 1,2).

The mean probing pocket depth preoperatively was 5.5±1.2 mm, which reduced to 3.13±0.99 mm at 3 months and showed a further reduction to 2.38±0.74 mm after 6 months. The mean reduction in probing depth from baseline to 3 months was 2.37±1.41 mm, which was statistically significant (P<0.05). The mean reduction from baseline to 6 months was 3.12±1.25 mm that was again statistically significant (P<0.05). A significant reduction in probing depth was also observed between the third and sixth months (0.75±0.46mm P<0.05). The mean probing attachment level at baseline was 6.75±1.67 mm. It showed a reduction to 4.75±1.67 mm and 4.00±1.6 mm at 3 months and 6 months respectively).This indicated an attachment gain of 2.00±1.31 mm at 3 months and 2.75±1.17 mm at 6 months compared to the baseline. This gain in attachment was statistically significant (P<0.05). A statistically significant attachment gain of 0.75±0.46 mm was also observed between 3 months and 6 months postoperatively. Preoperatively, the mean horizontal attachment level was 8.63±2.5 mm, as measured from the cementoenamel junction. It reduced to 5.88±1.81 mm at 3 months and 5.38±1.85 mm at 6 months postoperatively. The mean horizontal attachment gain at 3 months was 2.75±1.04 mm which was highly significant (P=0.01). At 6 months a 3.25±1.28 mm mean gain in horizontal attachment was noticed, which again was significant (P<0.05). Between 3 months and 6 months a significantly mean gain of 0.5±0.53 mm was observed. The mean gingival recession present at baseline was 1.25±1.04 mm. It showed an increase to 1.63±1.3 mm after 3 months, with a mean increase of 0.38±0.52 mm. But this increase was statistically insignificant (P>0.05). However, no change was observed between the third and the sixth months. Radiographs were used in the present study to supplement the clinical findings. Because of the anatomical constraints related to the furcation, quantitative analyses of the radiographs were not possible. However, visibly discernible changes were observed in majority of the radiographs after 6 months when compared to the baseline radiographs. A definite increase in radioopacity was observed in the furcation areas of mandibular grade II furcation defects at 6 months. Similar findings were noticed in the radiographs of maxillary grade II furcations also, but the changes were less apparent and could have been influenced by the presence of the palatal root.

## Discussion

Furcation involvements present a unique clinical situation in the practice of Periodontics in terms of diagnosis, management, and prognosis of the affected teeth. Most of the research on the management of furcation involvement has focused on grade II furcation defects because of the encouraging clinical results obtained with various regenerative procedures.

Bone replacement grafts constitute the most widely used therapeutic strategy for the correction of periodontal osseous defects. Hydroxyapatite graft materials have been commonly used as bone substitutes in the management of intrabony defects. Although alloplastic bone materials support periodontal repair rather than regeneration, on the basis of clinical outcome measures, they bring about similar extent of improvement compared to bone allografts. There is ample evidence to suggest that use of hydroxyapatite grafts in the treatment of intrabony defects result in increased bone level, gain in clinical attachment, and reduction in probing depth when compared to open flap debridement(3). However, there is a paucity of data regarding the use of hydroxyapatite alloplasts in the management of furcation defects.

In the present study, hydroxyapatite bone replacement material (Periobone G) was evaluated in 9 grade II furcation defects in molars. The uneventful healing observed during the post surgical period once again confirmed the biocompatibility of the graft material as shown in the earlier studies (7). By using a porous hydroxylapatite implant (Interpore 200), Kenney et al (5) had achieved a mean reduction of 2.08 mm in 23 grade II furcation defects. A meta-analysis of the studies, which evaluated bone grafts in grade II furcation defects, revealed a mean probing depth reduction of 1.9-2.31 mm in grafted sites and 0-1.8 mm in control sites(3).

When the mean gain in clinical attachment level was considered, the present study attained a significant gain of 2.75 mm at 6 months, which was comparatively better than that achieved by Kenney et al(5). In the meta-analysis of studies evaluating bone grafts, the mean gain in clinical attachment obtained with the use of bone grafts in grade II furcation defects ranges from 1.6 to 1.9 mm (3).

The horizontal attachment level in the present study was assessed using the calibrated color-coded Nabers probe as suggested by Eickholz et al (8). The cementoenamel junction was used as a fixed reference point for the measurements according to Yukna and Yukna (9). Using this method, the mean gain in horizontal attachment level after 6 months observed in this study was 3.25 mm. Similarly, Kenney et al (5) obtained a mean horizontal bone fill of 1.56 mm at six months reentry. The mean horizontal defect fill observed in general with bone grafts ranges from 1.6 to 3.4 mm according to the meta-analysis. However, the results of the present study cannot be directly compared with the previous studies, as open bone measurements were not used. The gain in attachment observed can either be due to bone fill or due to connective tissue attachment in the furcation that may present more resistance to the passage of the probe.

The mean gingival recession observed in the present study was 0.38 mm. Mean recession observed in the past studies with the use of bone grafts in grade II furcation defects ranges from 0.2 mm to 1.7 mm (3).

Intraoral periapical radiographs made with the long cone paralleling technique were used in the present study to supplement the clinical observations. Radiographs have a limited value in assessing the response to regenerative therapies in furcation defects, especially in the case of maxillary molars because of the superimposition of the palatal root. In grade II furcation defects, the presence of remaining bone in the interradicular aspect may also to some extent influence the radiographic picture. A clear increase in radioopacity in the furcation area was observed in the radiographs of mandibular molars at 6 months when compared to the baseline radiographs. This may be indicative of bone fill occurred in the furcation area, as these teeth showed a change in clinical furcation grade from grade II to grade I. Although not as apparent as in the case of mandibular molars, the radiographs of two maxillary molars exhibited an increased radioopacity at the furcation aspect.

Based on the observations of the present study, it can be concluded that porous hydroxyapatite alloplasts are effective in reducing pocket depth and bringing out gain in attachment levels when used in the management of grade II furcation defects. However, further long-term controlled studies involving larger sample sizes are required for determining predictable outcomes of the material on a long-term basis, when used in furcation defects.
